# Supplementary material for: Evidence from three taxonomically distinct species for a non-AhR mechanism of developmental neurotoxicity of an environmentally derived mixture of polycyclic aromatic hydrocarbons
Source: bioRxiv. 2026 Aug 18:2026.08.12.743995. Preprint. [Version 1] doi: 10.64898/2026.08.12.743995 (PMC13587152; doi:10.64898/2026.08.12.743995)
Supplement: Supplementary file 1 [file media-1.pdf]

A

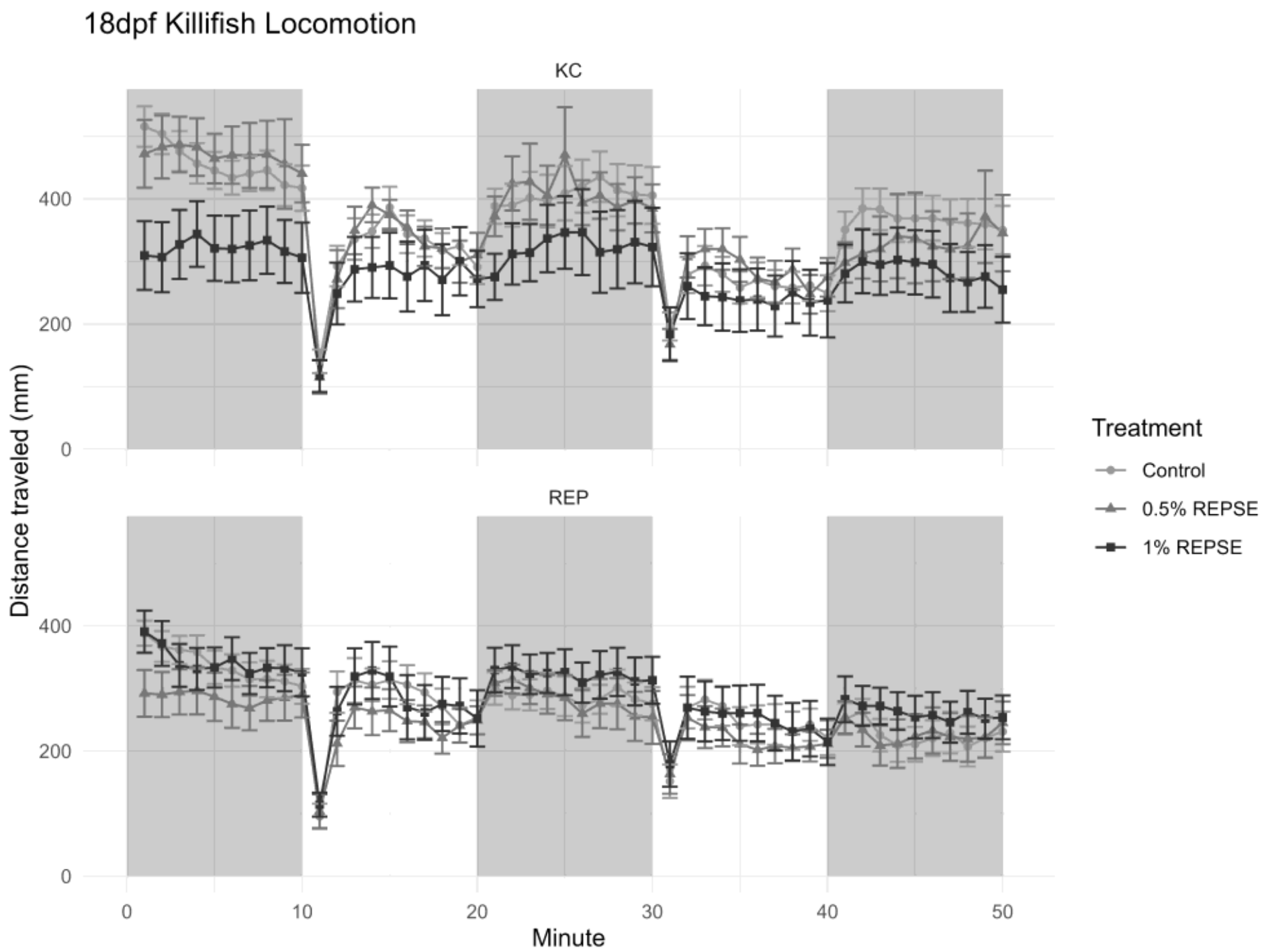

B

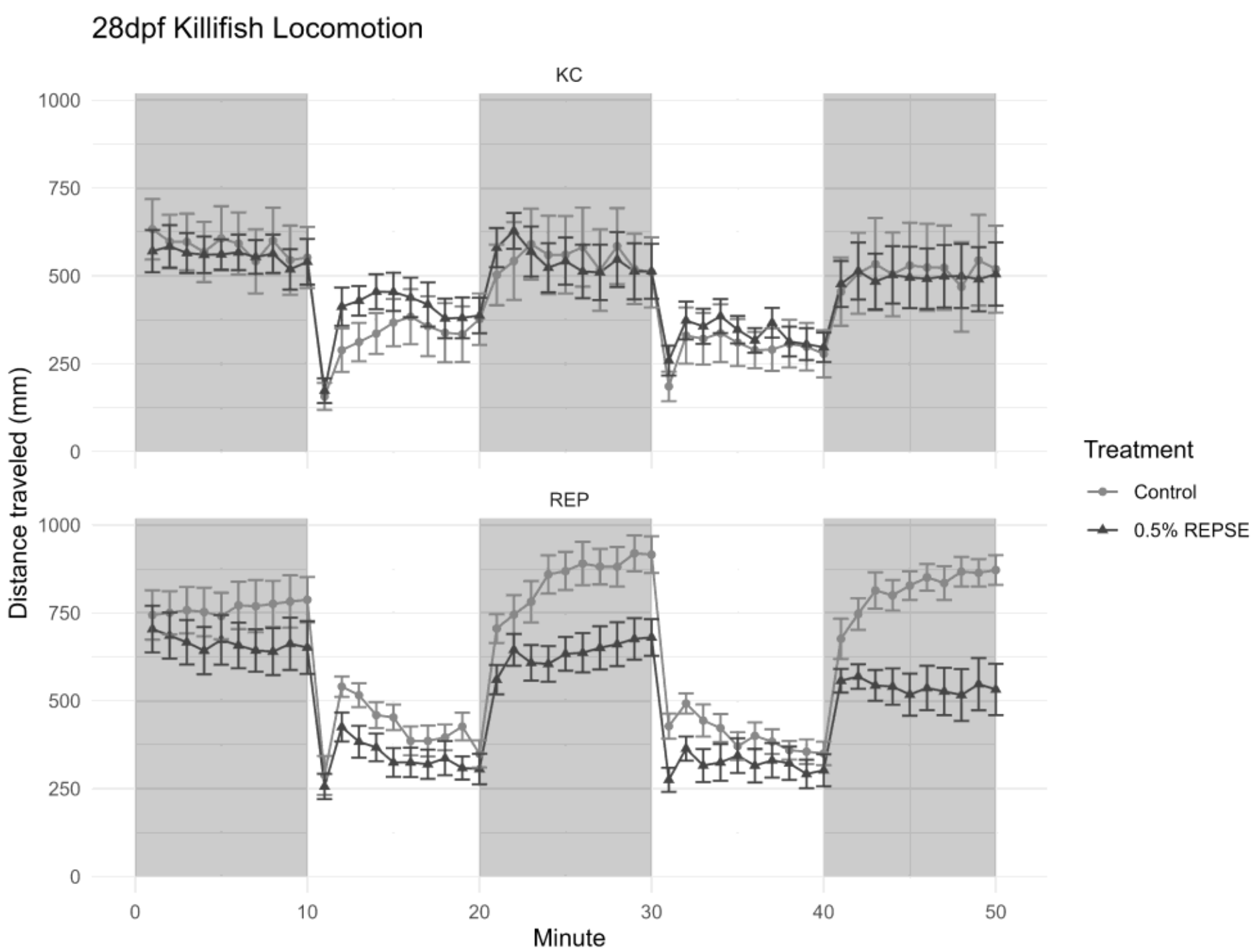

Figure S1. Killifish Neurobehavior. Minute-by-minute traces of distance traveled per minute by **A)** 18dpf and **B)** 28dpf killifish larvae from KC (upper graph) and REP (lower graph). Background shading indicates light/dark status of chamber. Points and error bars represent mean +/- SEM for each treatment group.

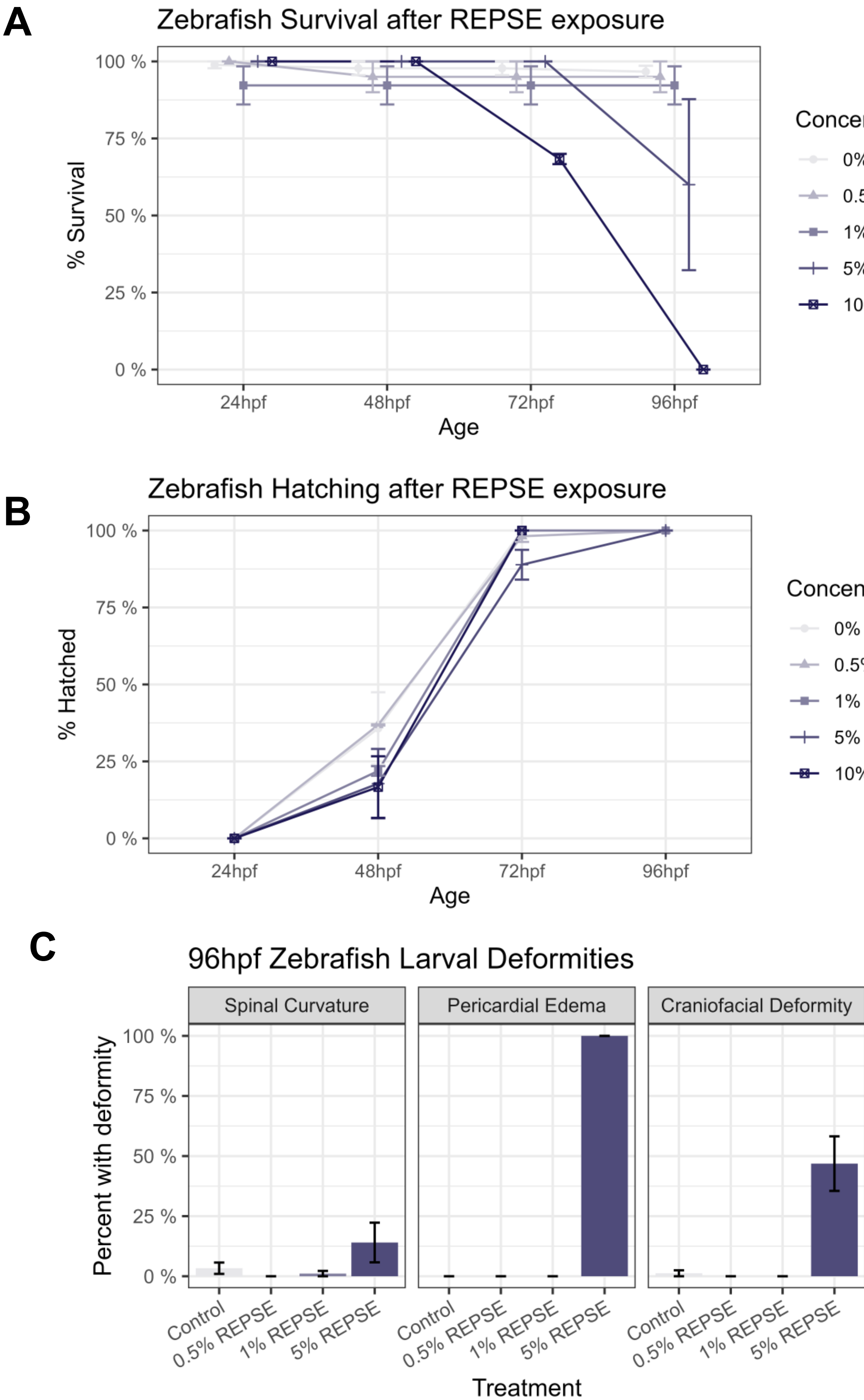

Figure S2. Zebrafish Survival, hatching, and deformities after REPSE exposure. Daily survival **(A)** and hatching rates (proportion of surviving individuals hatched) **(B)** for zebrafish larvae from 24-96hpf. Points and error bars represent mean +/- SEM for each treatment group, across 3-4 biological replicates. **C)** Deformity rates by treatment group for frequently observed deformities at 96hpf.

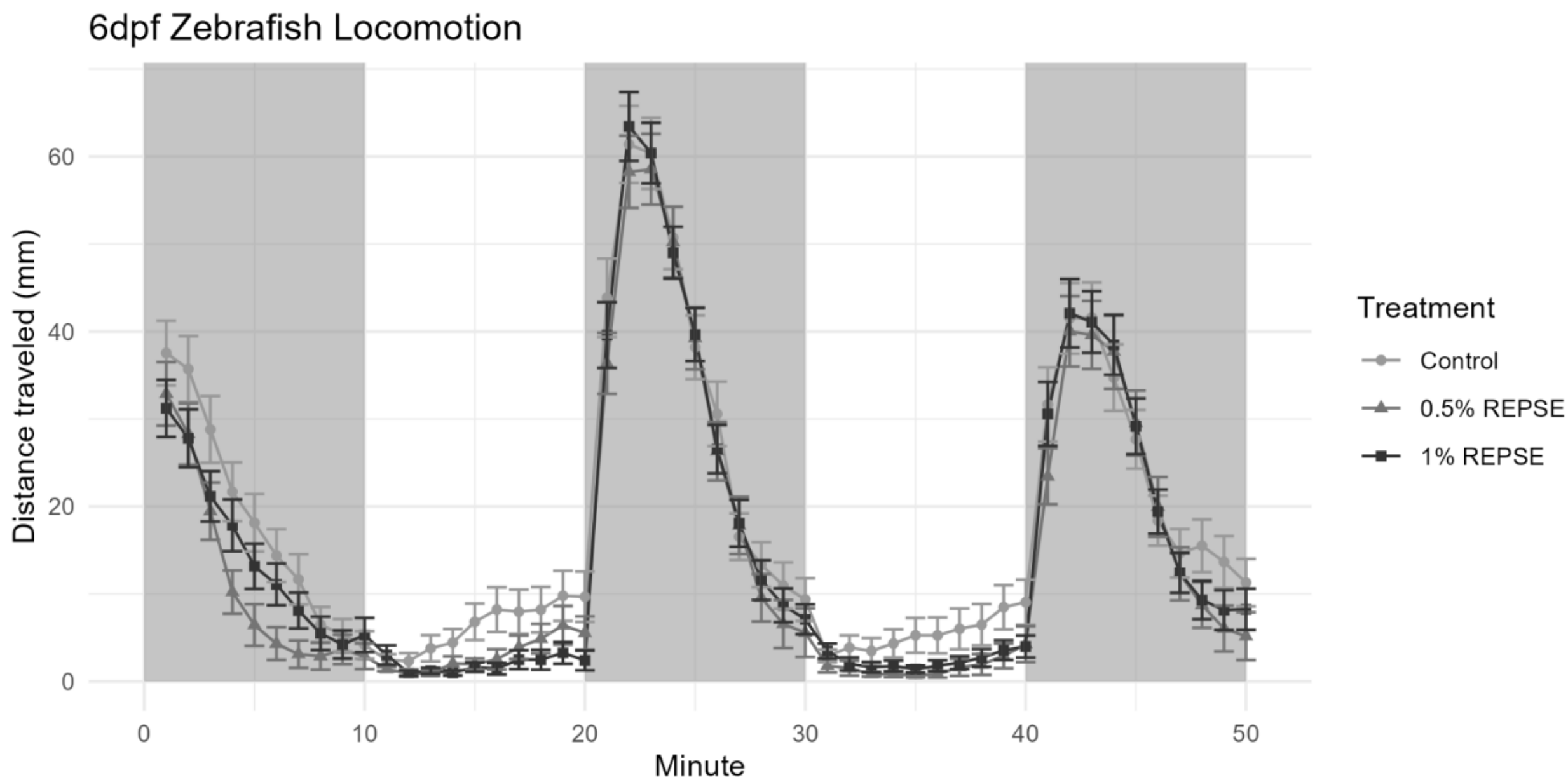

Figure S3. Zebrafish Neurobehavior. Minute-by-minute traces of distance traveled per minute. Background shading indicates light/dark status of chamber. Points and error bars represent mean +/- SEM for each treatment group.

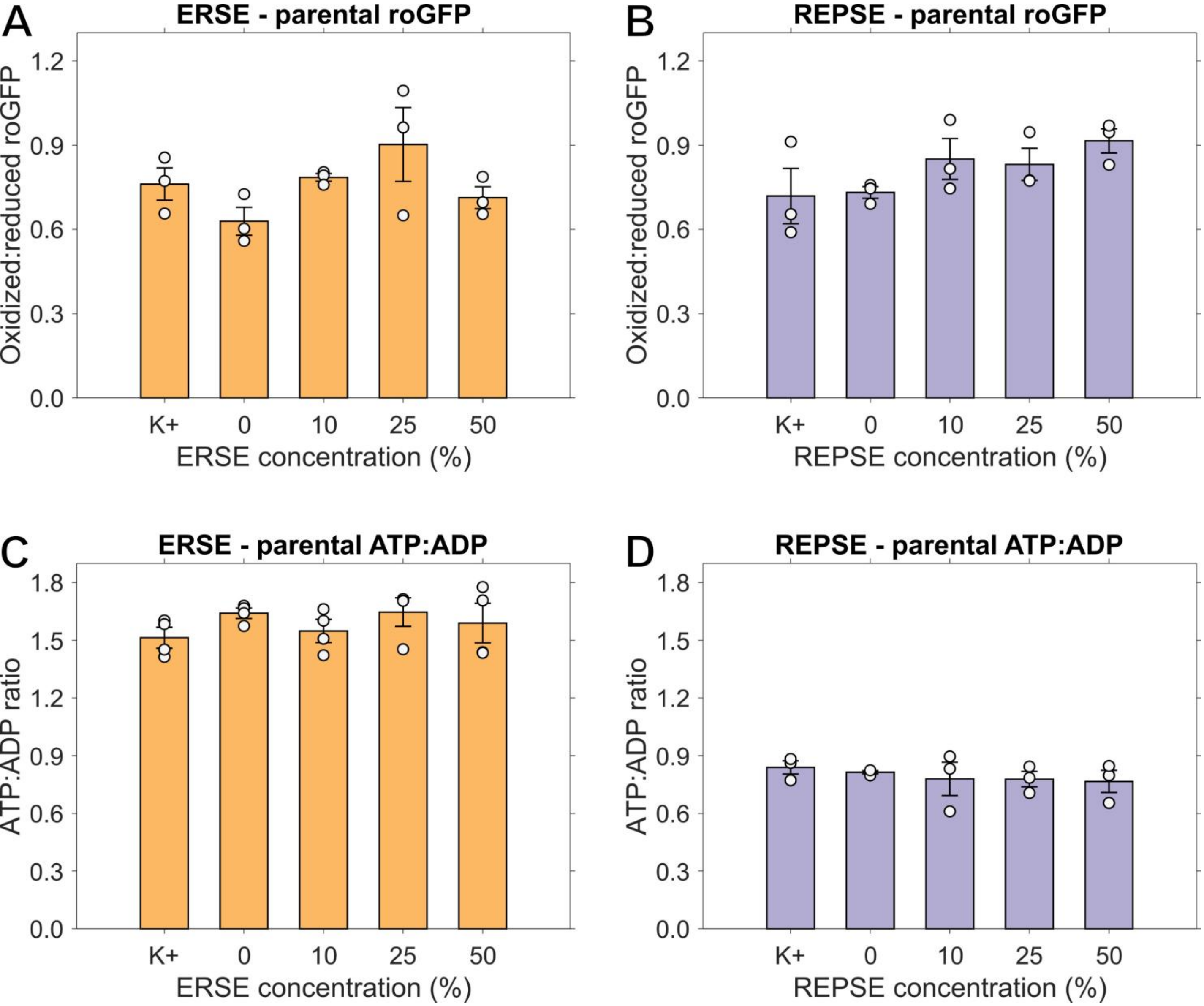

Figure S4. ***C. elegans*** **redox state and ATP levels after parental exposure to ERSE/REPSE.** Oxidized to reduced roGFP ratio in cephalic dopaminergic neurons of offspring of worms exposed to **A)** ERSE and **B)** REPSE. ATP to ADP ratio in cephalic dopaminergic neurons of offspring of worms exposed to **C)** ERSE and **D)** REPSE. N = 3-4 biological replicates, n = 30-40 neurons per replicate per concentration, one-way ANOVA with Dunnett's post hoc test.

TABLE S1. Concentrations of PAHs from Republic Sediment Extract (REPSE). Values represent averages (ng/mL) of two replicate aliquots of REPSE. Those concentrations below the minimum detection limit are represented by <MDL.

Compound	Concentration (µg/L)
Total PAHs	***746.70***
Fluoranthene	136.89
Pyrene	96.08
Phenanthrene	85.65
Acenaphthene	47.05
1,2-Benzanthracene	40.40
1,2-benzofluorene	37.80
Anthracene	32.83
Fluorene	26.48
Chrysene	24.77
Benzo(b)fluoranthene	22.85
Benzo(a)pyrene	19.95
Benzo(e)pyrene	22.01
Benzo(k)fluoranthene	20.08
Benzo(a)fluoranthene	16.70
Dibenzofuran	14.05
2-methylphenanthrene	14.29
Carbazole	11.65
Naphthalene	10.89
Benzo(b)chrysene	9.12
1-methylphenanthrene	8.69
Indeno(1,2,3-c,d)pyrene	7.39
Benzo(c)phenanthrene	8.44
Dibenzothiophene	6.12
Dibenzo(a,l)pyrene	5.93
Perylene	4.52
Dibenzo(a,j)anthracene	4.57
Acenaphthylene	2.56
2,6-dimethylnaphthalene	2.85
Dibenzo(a,h)anthracene	2.54
Picene	1.97
1-methylnaphthalene	1.00
Benzo(g,h,i)perylene	0.52
3-methylcholanthrene	<MDL
Retene	<MDL
Pentachlorophenol	<MDL
Tetrachloro-1,4-benzoquinone	<MDL
